# Quantifying the integrated physiological effects of endothelin-1 on cardiovascular and renal function in healthy subjects: a mathematical modeling analysis

**DOI:** 10.3389/fphar.2024.1332394

**Published:** 2024-04-05

**Authors:** Hongtao Yu, Peter Greasley, Hiddo Lambers-Heerspink, David W. Boulton, Bengt Hamrén, K. Melissa Hallow

**Affiliations:** ^1^ Clinical Pharmacology and Quantitative Pharmacology, Clinical Pharmacology and Safety Sciences, R&D, AstraZeneca, Gaithersburg, MD, United States; ^2^ Early Clinical Development, Research, and Early Development, Cardiovascular, Renal and Metabolism, BioPharmaceutical R&D, AstraZeneca, Gothenburg, Sweden; ^3^ Department of Clinical Pharmacy and Pharmacology, University of Groningen, Groningen, Netherlands; ^4^ The George Institute for Global Health, Sydney, NSW, Australia; ^5^ Clinical Pharmacology and Quantitative Pharmacology, Clinical Pharmacology and Safety Sciences, R&D, AstraZeneca, Gothenburg, Sweden; ^6^ School of Chemical, Materials, and Biomedical Engineering, University of Georgia, Athens, GA, United States; ^7^ Department of Epidemiology and Biostatistics, University of Georgia, Athens, GA, United States

**Keywords:** endothelin-1, natriuresis and diuresis, ET_A_ receptor antagonist, ET_B_ receptor antagonist, cardiovascular and renal function, mathematical modeling

## Abstract

Endothelin-1 (ET-1) is a potent vasoconstrictor with strong anti-natriuretic and anti-diuretic effects. While many experimental studies have elucidated the mechanisms of ET-1 through its two receptors, ET_A_ and ET_B_, the complexity of responses and sometimes conflicting data make it challenging to understand the effects of ET-1, as well as potential therapeutic antagonism of ET-1 receptors, on human physiology. In this study, we aimed to develop an integrated and quantitative description of ET-1 effects on cardiovascular and renal function in healthy humans by coupling existing experimental data with a mathematical model of ET-1 kinetics and an existing mathematical model of cardiorenal function. Using a novel agnostic and iterative approach to incorporating and testing potential mechanisms, we identified a minimal set of physiological actions of endothelin-1 through ET_A_ and ET_B_ receptors by fitting the physiological responses (changes in blood pressure, renal blood flow, glomerular filtration rate (GFR), and sodium/water excretion) to ET-1 infusion, with and without ET_A_/ET_B_ antagonism. The identified mechanisms align with previous experimental studies on ET-1 and offer novel insights into the relative magnitude and significance of endothelin’s effects. This model serves as a foundation for further investigating the mechanisms of ET-1 and its antagonists.

## 1 Introduction

ET-1 is a potent vasoconstrictor, especially in the renal vasculature, and is anti-natriuretic and anti-diuretic. It exerts these effects through its two receptors–ET_A_ and ET_B_. Both receptors have been detected in all tissues with blood supply, indicating their ubiquitous expression (Regard et al., 2008; Davenport et al., 2016). Their relative and absolute densities vary by location and across species. Systemically, saturation binding assays show that resistance vessels express primarily ET_A_, while in the kidney, relative expression of ET_B_ overall is much higher compared to ET_A_ (Davenport et al., 2016). Within the kidney, though, the relative concentrations of ET_A_ and ET_B_ vary. ET_A_ and ET_B_ have both been found to be expressed in the preafferent, afferent, efferent, and peritubular capillaries, as well is in the proximal tubule, thick ascending limb, and collecting duct. But preafferent and afferent arterioles have relatively higher expression of ET_A_, while efferent and peritubular arterioles have higher expression of ET_B_. Both receptor types are also expressed in the tubule. ET_A_ is found primarily in the proximal tubule. ET_B_ is found in all segments, but the inner medullary collecting duct has the highest density of ET_B_ receptors (Kohan et al., 2011).

A large body of experimental studies have provided a great deal of data for understanding of the effects of ET-1 through each receptor by utilizing various approaches, including ET-1 infusion studies, knock-out studies, and perturbation with various receptor agonists/antagonists [for a thorough review, see (Davenport et al., 2016; Kohan et al., 2011)]. However, the complexity of responses and sometimes conflicting data, especially across species, make it challenging to predict effects in human physiology. For instance, while it is well established that ET-1 causes vasoconstriction through ET_A_, the effects of ET_B_ are more complex. Both ET_B_ agonism and antagonism have been shown to cause vasoconstriction (Haynes et al., 1995; Love et al., 2000). ET_B_ appears to constrict the afferent arteriole but dilate the efferent arteriole (Inscho et al., 2005). In addition, while ET-1 infusion certainly exerts anti-natriuretic and anti-diuretic effects, under some conditions ET-1 appears to inhibit reabsorption and promote natriuresis/diuresis in the collecting duct (Kohan et al., 2011).

Mathematical modeling can be a tool for integrating knowledge of physiology and various data sets into a consistent quantitative framework in order to better understand a system. In this study, we aimed to utilize existing experimental data to develop an integrated and quantitative description of endothelin effects on cardiovascular and renal function in healthy humans. Using a mathematical model of endothelin kinetics published in a sister paper, coupled to an existing mathematical model of cardiorenal function (Hallow et al., 2014; Hallow and Gebremichael, 2017; Hallow et al., 2018), we aimed to estimate the magnitude of physiological actions of endothelin-1 through ET_A_ and ET_B_ receptors by fitting the physiological response to ET-1 infusion, with and without ET_A_/ET_B_ antagonism. Quantitively understanding the physiological effects of ET-1 and ET-1 antagonism in normal subjects is a first step toward better understanding its role in cardiovascular and renal disease, and both the beneficial effects and deleterious fluid retention in previous clinical studies of ET_A_ antagonists. This knowledge could help harness ET_A_ antagonists to gain renal benefit while mitigating fluid retention.

## 2 Materials and methods

### 2.1 Cardiorenal model

**FIGURE 1:**
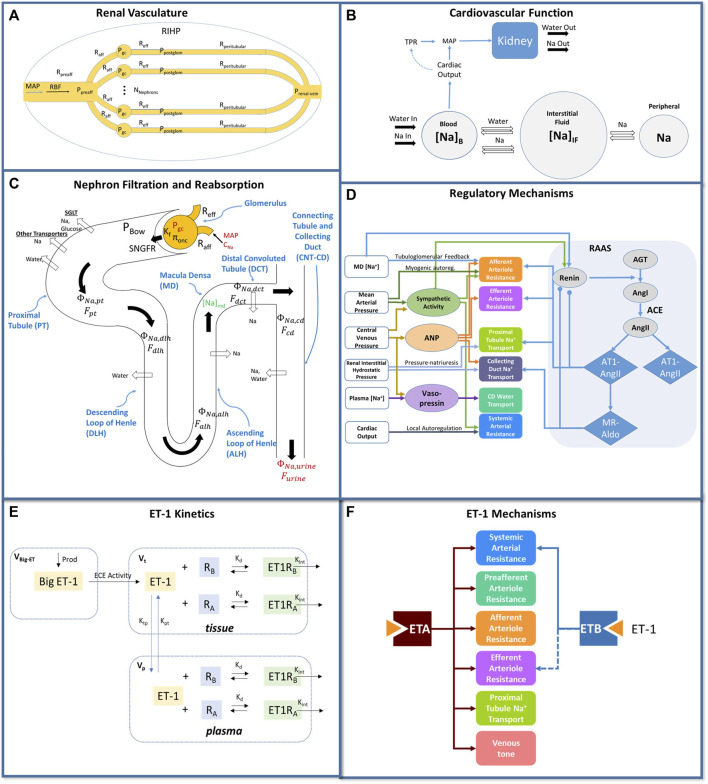
Mathematical model of cardiorenal function. **(A)** the renal vasculature is modeled by a single preafferent resistance vessel flowing into N parallel nephrons with an afferent, efferent, and peritubular resistance; RBF and glomerular hydrostatic and oncotic pressures are calculated as a function of MAP, renal venous pressure, and resistances. **(B)** The balance between Na^+^ and water excretion and intake determines blood volume and plasma Na^+^ concentration. Na^+^ and water move between the blood and interstitial fluid according to starling forces, and Na^+^ may be sequestered non-osmotically in a peripheral storage compartment. Blood volume and venous compliance and capacitance determines venous return and cardiac output, which together with total peripheral resistance, determine MAP. **(C)** Glomerular filtration is described by the balance of starling forces and the glomerular ultrafiltration coefficient K_f_. Na^+^, glucose, and water are reabsorbed at different fractional rates in the proximal tubule, loop of Henle, distal convoluted tubule, and connecting tubule/collecting duct. **(D)** Multiple regulatory mechanisms, including the renin-angiotensin-aldosterone system (RAAS), renal sympathetic activity, atrial natriuretic peptide (ANP), and vasopressin, provide feedback on model variables. **(E)** Endothelin-1 kinetics submodel. Big ET-1 is assumed to be produced at a constant rate; ECE converts Big ET-1 to ET-1 in the tissue compartment; ET-1 is distributed between the tissue and plasma compartments; in each compartment, ET-1 binds to ET_A_ and ET_B_ receptors to form receptor-ligand complexes which are then cleared by internalization. **(F)** Physiological effects of ET-1 through the ET_A_ and ET_B_ receptor, included in the final model. P, pressure; R, resistance; RBF: renal blood flow; MAP, mean arterial pressure; RIHP, renal interstitial hydrostatic pressure; Na, sodium; SNGFR: single nephron glomerular filtration rate; ϕ, mass flow rate; F, volumetric flow rate; C, concentration; MD, macula densa; ANP, atrial natriuretic peptide; RAAS, renin angiotensin aldosterone system; AGT, angiotensinogen; Ang, angiotensin; AT1, angiotensin receptor type 1; AT2, angiotensin receptor type 2; MR, mineralocorticoid receptor; aldo, aldosterone; V, volume; k_d_, binding affinity; k_tp_ and k_pt_, intercompartmental transfer rate constants.

We utilized a previously published cardiorenal model (Hallow et al., 2014; Hallow et al., 2017; Hallow and Gebremichael, 2017; Hallow et al., 2018), summarized schematically in Figures 1A–D. This model describes the key physiological processes of kidney function, Na^+^ and water homeostasis, and blood pressure control, including blood flow and pressure through the renal vasculature (Figure 1A); renal filtration, reabsorption, and excretion of sodium, water, and glucose (Figure 1C); whole-body fluid/electrolyte distribution (Figure 1B); and key neurohormonal and intrinsic feedback mechanisms (Figure 1D). Full model equations, parameters, and initial conditions have been published previously.

### 2.2 Endothelin 1 kinetics model

The development, calibration, and validation of a mathematical model of endothelin-1 kinetics is described in a sister paper (Hallow et al., manuscript in review - Frontiers in Pharmacology), and illustrated schematically in Figure 1E. In brief, Big ET-1 is assumed to be produced at a constant rate; ECE converts Big ET-1 to ET-1 in the tissue compartment; ET-1 is distributed between the tissue and plasma compartments; in each compartment, ET-1 binds to ET_A_ and ET_B_ receptors to form receptor-ligand complexes which are then cleared by internalization. The model also describes competitive binding of antagonists to the ET_A_ and ET_B_ receptor, and allows specification of selectivity and binding affinities for each receptor. The model was calibrated to the response to infusion of ET-1 or BigET-1 in three studies (Kaasjager et al., 1997; Parker et al., 1999; Hunter et al., 2017), and was validated by reproducing the ET-1 response to ET-1 in a different study (Bohm et al., 2003), as well as the ET-1 response to ET_A_ antagonist BQ123 and ET_B_ antagonist BQ788.

### 2.3 Integration and calibration of endothelin-1 effects in the cardiorenal model

The model of endothelin-1 kinetics and receptor antagonism was incorporated into and mechanistically linked with the cardiorenal model. Specifically, endothelin-1 exerts its physiological effects by binding to ET_A_ and ET_B_ receptors. Thus, the concentrations of ET-1 bound to ET_A_ or ET_B_ receptors [(ET1R_A_) and (ET1R_B_), respectively in Figure 1E] were linked to the mechanistic effects of each receptor.

To do this, it was first necessary to identify the primary mechanisms of each receptor, and then to determine the shape and magnitude of the mathematical relationship between each ET1-receptor complex and its mechanisms, as presented in Figure 1F.

**FIGURE 2:**
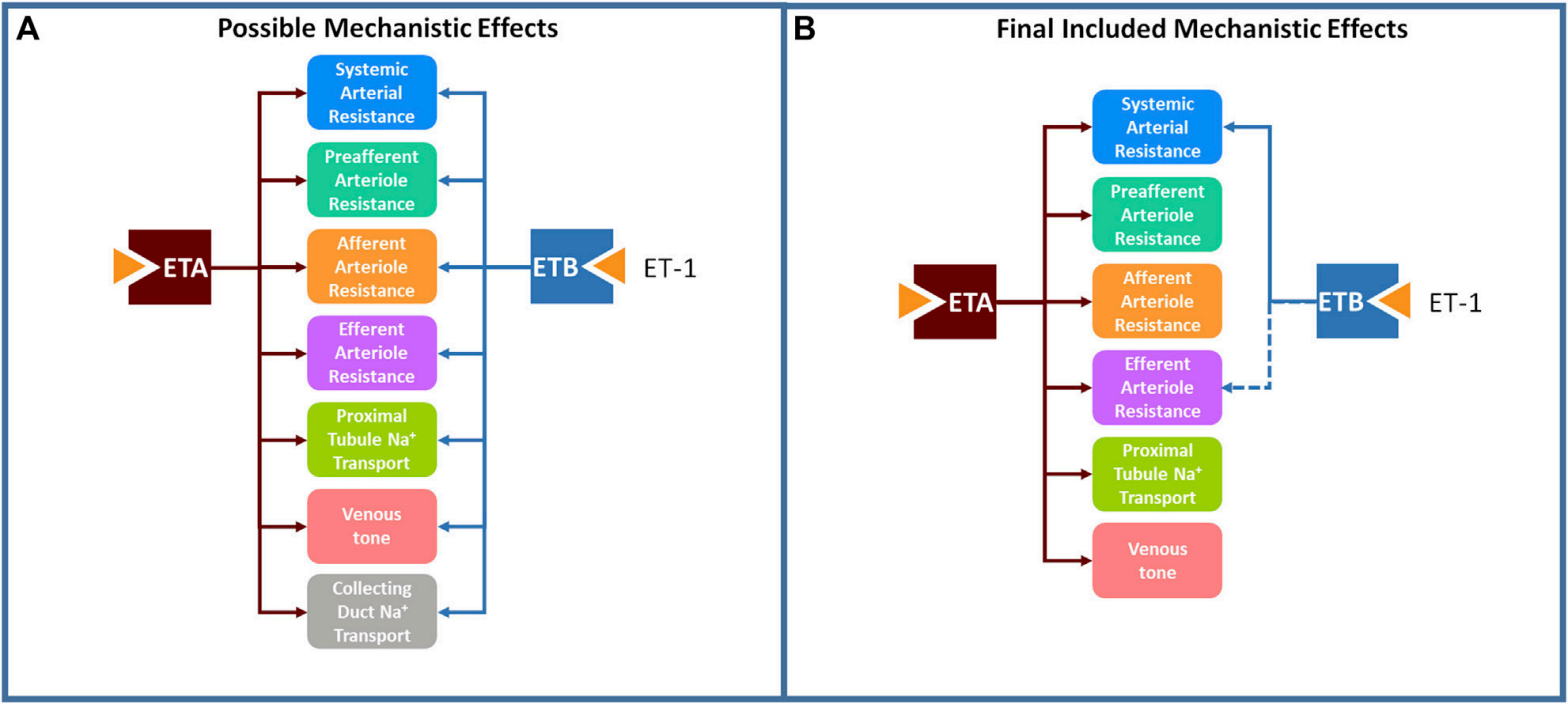
A large set of postulated mechanistic effects of ET-1 through ET_A_ and ET_B_ tested for inclusion in the model **(A)**, and a subset of these mechanisms, found to be necessary to explain experimental data, were included in the final model **(B)**.

Based on the body of available experimental data (Haynes et al., 1995; Love et al., 2000; Inscho et al., 2005; Kohan et al., 2011; Davenport et al., 2016), we postulated possible mechanistic effects of ET-1 through the ET_A_ and ET_B_ receptor, illustrated in Figure 2A. However, we took an agnostic approach to the existence, magnitude, and functional form of each relationship. Most physiological effects are saturable and thus well described as sigmoidal when considered over the full range of concentrations. However, if the range of concentrations observed physiologically or experimentally do not sufficiently cover the extremes, the saturation may not be detectable. Also, even if saturation occurs, there is not always sufficient data to estimate both the magnitude and steepness of the relationship. In these cases, a linear model, which only requires estimation of the slope m, may be more appropriate. Thus, for each possible mechanism, two possible functional forms were considered: linear (Eq. 1) and sigmoidal (Eq. 2).

$$E_{linear}=\max\left(\;1+m_{i}\left(\left[ET1R_{i}]-[ET1R_{i0}\right]\right),0\right)$$ (1)

$$E_{sig}=1+\frac{m_{i}}{1+e^{\frac{\left[ET1R_{i}\right]-\left[ET1R_{i0}\right]}{b}}}-\frac{m_{i}}{2}$$ (2)

Here, ET1R_i_ represents the concentration of ET-1 bound to the either the ET_A_ or ET_B_ receptor. ET1R_i0_ is the bound concentration under normal conditions. *m*_*i*_ defines the magnitude of the effect, and for the sigmoidal response, *b* defines the steepness of the sigmoidal function. E is the physiological effect on the target parameter. E is one when ET1R_i_ is at its normal concentration, and may increase or decrease the target parameter as ET1R_i_ changes.

#### 2.3.1 Mechanism selection

**FIGURE 3:**
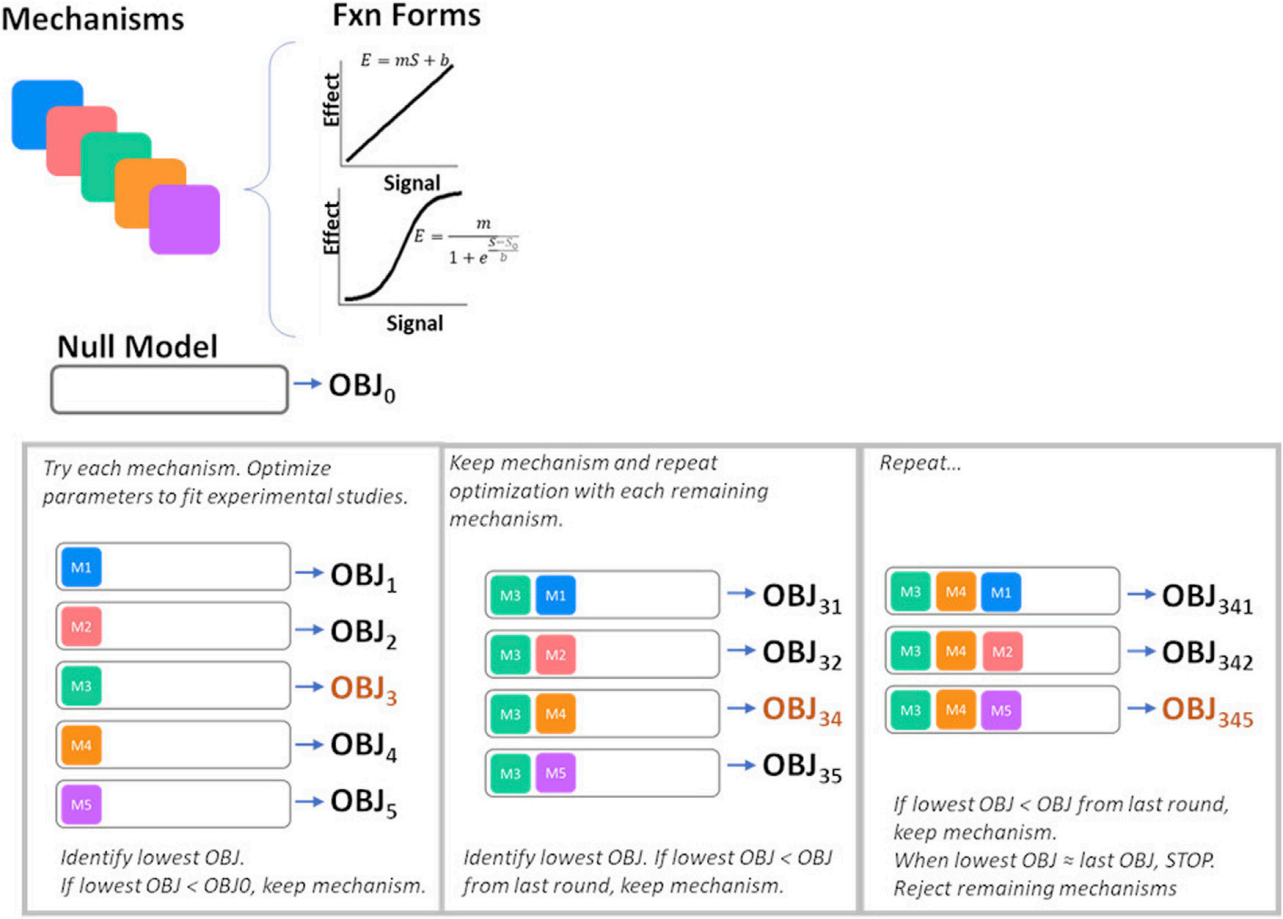
Process for mechanism selection and parameter estimation. OBJ, objective function value.

The possible mechanistic effects of ET-1 through the ET_A_ and ET_B_ receptor, illustrated in Figure 2A, were first tested and selected for inclusion in the final model using a forward selection approach followed by a backward elimination step. The mechanism selection process is illustrated in Figure 3. Briefly, the base model, referred to as the NULL model, contained no mechanistic effects of ET-1. An initial objective function (OBJ) was determined by calculating the sum of the square error between the simulation and observed data for two experimental studies, described below. In the first round of selection, each mechanism and functional form was tested individually. For each, the slope *m* (linear) or slope *m* and steepness *b* (sigmoidal) was optimized to the experimental data. The mechanism that produced the greatest OBJ reduction, compared to the NULL model, was kept in the model for the next round. In the second round, each remaining mechanism/shape combination was tested in combination with the mechanism from the first round. The mechanism that produced the greatest reduction in OBJ, compared to the first round OBJ, was kept for the next round. This was repeated until no further improvements in OBJ occurred. At this point, the remaining mechanisms that did not improve OBJ were considered unimportant in explaining the experimental data, and were not included in the model. For the mechanisms identified as important in each of the forward rounds, a backward elimination round was used to confirm the contribution of each included mechanism. For this, first the OBJ with all included mechanisms was calculated. Then the OBJ was calculated after dropping each of the mechanisms individually. If any mechanism did not increase OBJ when dropped, this would indicate that that mechanism was not necessary to explain the data.

#### 2.3.2 Parameter estimation

During the mechanism selection process, unknown model parameters were estimated by simultaneously fitting two experimental studies. These two studies were selected because they were conducted in human subjects and measured both plasma ET-1 and renal and systemic responses over time. The studies provide complementary information for constraining model parameters.

*Infusion of increasing doses of ET-1:* In (Kaasjager et al., 1997), six healthy subjects were placed on a diet of 200 mmol sodium per day for 5 days. They were then administered an infusion of ET-1 at increasing infusion rates: 0.5 ng/kg/min (0.2 pmol/kg/min) ET-1 for 60 min, followed by 1 ng/kg/min (0.4 pmol/kg/min) for 60 min, followed by a final 2.0 ng/kg/min (0.8 pmol/kg/min) for 60 min. Subjects were given an oral water load of 25 mL/kg body weight before the experiment began, and were asked to drink water matching their urinary output volume to maintain water loading. Plasma ET-1 was measured before infusion and at 75, 125, and 225 min after the start of the infusion. GFR was measured through inulin clearance and estimated renal plasma flow (RPF) was measured through para-aminohippuric acid (PAH). Renal blood flow (RBF) was calculated as RPF*(1- packed cell volume). Mean arterial pressure (MAP) was measured continuously. Renal vascular resistance (RVR) was calculated as MAP/RBF. Urine was collected throughout the study and urine flow rate, sodium excretion rate, fractional excretion of sodium, and fractional excretion of lithium were reported.
ET_A_ or ET_B_ inhibition followed by ET-1 infusion: In Bohm et al. (2003), six healthy, male subjects were studied on three different days separated by at least 1 week. Subjects were infused with either 0.9% saline (for 15 min), the ET_A_ inhibitor BQ123 (2.5–5 nmol/kg/min for 50 min), or the ET_B_ inhibitor BQ788 (4 nmol/kg/min for 15 min). After 30 min, subjects were also infused with ET-1 (4 pmol/kg/min; 10 ng/kg/min) for 20 min. Plasma ET-1 was measured at 0, 15, 30, 40, and 50 min. RBF was measured through PAH clearance. MAP was measured continuously, and RVR was calculated from RBF and MAP.

Study protocols were simulated as described in each manuscript, including sodium and water loading, doses of ET-1, ET_A_, and ET_B_ antagonist administered, and timing of doses. Parameters were estimated by minimizing the least square error between the observed and model-predicted responses.

#### 2.3.3 Validation

The model was validated by simulating a separate experimental study of ET_A_ inhibition followed by ET-1 infusion (Vuurmans et al., 2004). In this study, nine healthy, male subjects were studied on four different days separated by at least 1 week, in randomized order. To maintain diuresis, subjects were infused with a 5% glucose solution, and then were instructed to consume water matching urinary output. Subjects then received either 0.9% saline (for 15 min) or the ET_A_ inhibitor VML588 at a dose of 0.05, 0.2, or 0.4 mg/kg/hr through the remainder of the study. Ninety minutes after the start of the study, subjects were also infused with ET-1 (1 pmol/kg/min) for 20 min. GFR was measured through inulin clearance and estimated renal plasma flow (RPF) was measured through para-aminohippuric acid (PAH). Renal blood flow (RBF) was calculated as RPF*(1- packed cell volume). Mean arterial pressure (MAP) was measured continuously. Renal vascular resistance (RVR) was calculated as MAP/RBF. Urine was collected at 30 min intervals and sodium excretion rate was reported.

#### 2.3.4 Technical implementation

The model was implemented in R v4.1.2 using the RxODE package (Wang et al., 2016). Optimization was performed using the L-BFGS-B method in the optim package. Model code is available at https://bitbucket.org/cardiorenalmodel/endothelin-dynamics.

## 3 Results and discussion

### 3.1 Model calibration and mechanism selection

Figure 2B shows the final mechanisms selected for inclusion in the model. Estimated parameter values are given in Table 1. For all mechanisms, a linear form was found to be sufficient, and use of a sigmoidal function did not improve the objective function. This should not be interpreted to mean that the relationships are not saturable - only that they are reasonably approximated as linear over the range of the available experimental data. There certainly must be saturation of effects at high concentrations. It may be that the concentrations in the experimental studies do not reach concentrations sufficient to saturate the response, or that the data is not sufficiently granular to detect nonlinearity.

**TABLE 1:** Estimated slope for each include mechanism, and contribution of mechanism to improvement in objective function.

Signal	Effect	Initial calibration			Refined calibration
		Slope (SE)	OBJ		
			Reduction from NULL (%)	Reduction from previous round (%)	
ET1-ET_A_	Preafferent Arteriole Resistance	0.344 (9.1%)	−59	−59	0.288 (8.9%)
	Proximal Tubule Na^+^ Reabsorption	0.041 (4.6%)	−18	−45	0.0311 (5.1%)
	Afferent Arteriole Resistance	1.79 (3.6%)	−2.3	−13	1.66 (3.5%)
	Systemic Arterial Resistance	0.068 (3.1%)	−4.2	−19	0.060 (3.5%)
	Efferent Arteriole Resistance	0.086 (12%)	−1.4	−10	0.0635 (14%)
ET1-ET_B_	Efferent Arteriole Resistance	−0.008 (19%)	−0.05	−4	−0.0059 (22%)
	Systemic Arterial Resistance	0.013 (5.1%)	−3.1	−19	0.0135 (5.2%)

**FIGURE 4:**
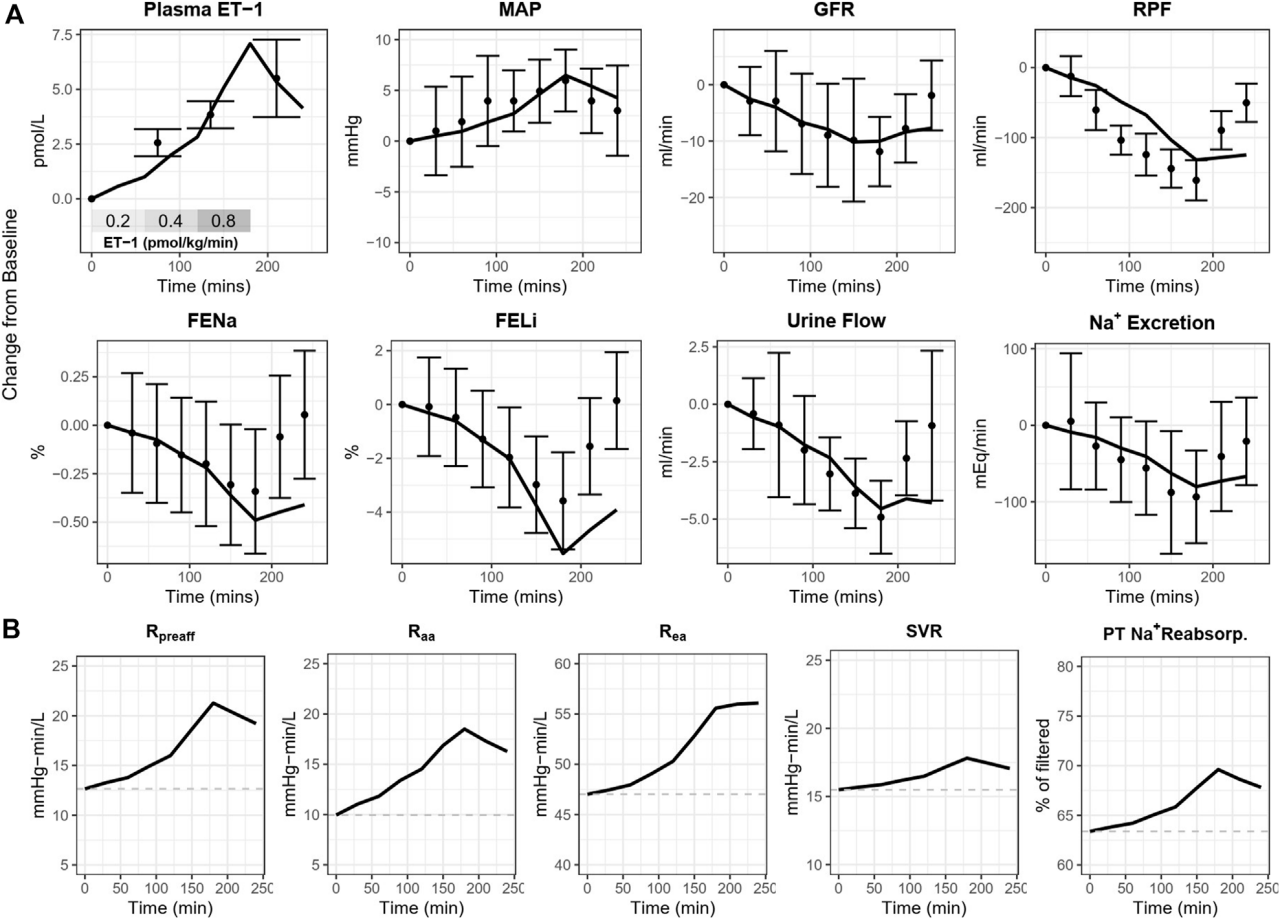
**(A)** Calibrated model reproduces the response of healthy subjects to ET-1 infusion observed in Kaasjager et al. (1997). **(B)** Simulated direct mechanistic effects of ET-1 infusion. MAP, mean arterial pressure; GFR, glomerular filtration rate; RPF, renal plasma flow; FENa, fractional excretion of sodium; FELi, fractional excretion of lithium; R, resistance; aa, afferent arteriole; ea, efferent arteriole; SVR, systemic vascular resistance; PT, proximal tubule.

**FIGURE 5:**
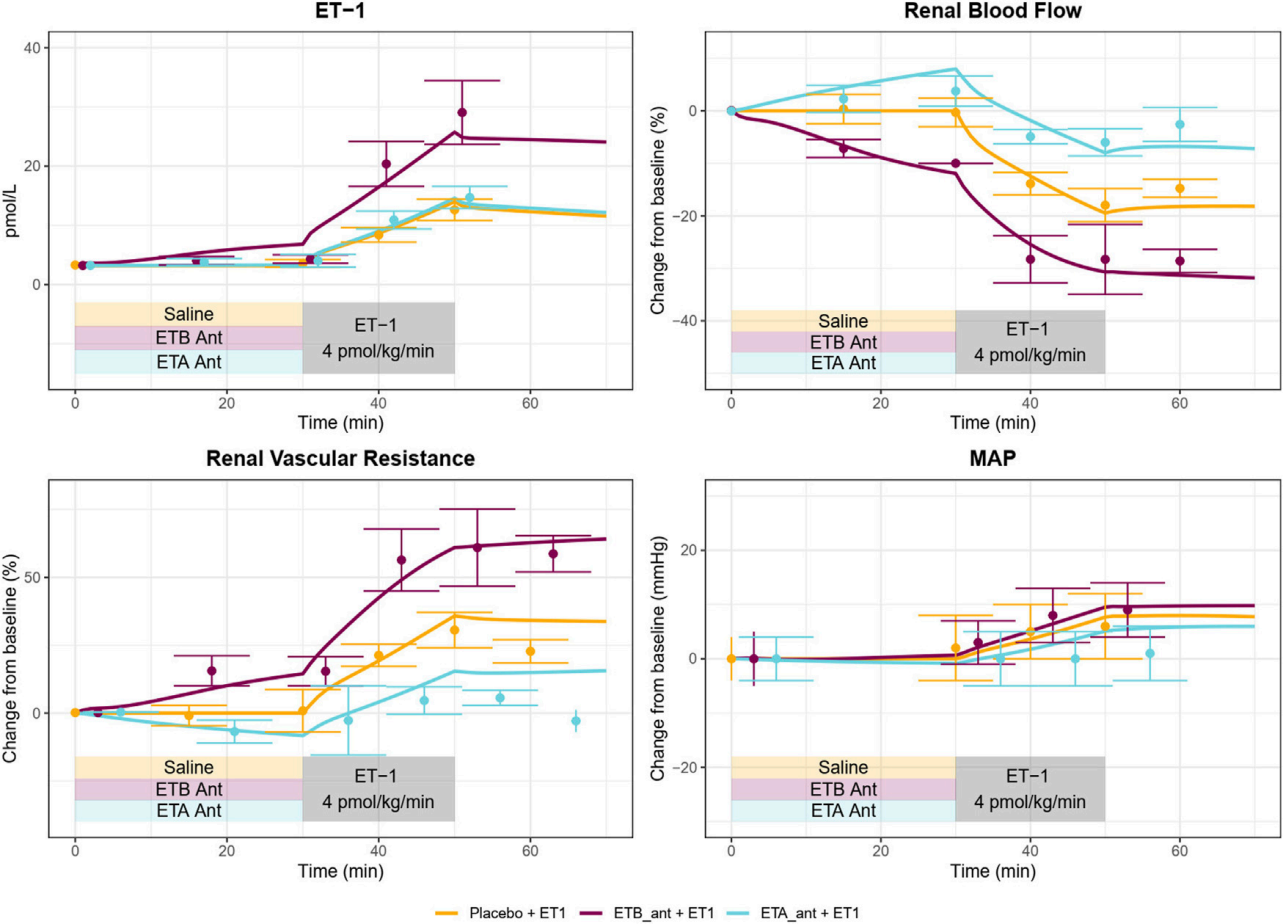
A Calibrated model reproduces the response of healthy subjects to ET_A_ or ET_B_ antagonism followed by ET-1 infusion observed in Bohm et al. (2003).

As shown in Figures 4A, 5, the calibrated model reasonably reproduced the observed magnitude and time course of changes in physiological variables in both experimental studies used for model calibration. The model was able to describe all of the key features of the response to ET-1 infusion (Figures 4A, 5—yellow), as well as the differing effects of ET_A_ and ET_B_ antagonism (Figures 5 – blue and purple). As observed in the experimental data, each antagonist alone had a minimal effect on RBF, RVR, and MAP, but blunted (ET_A_ antagonist) or exacerbated (ET_B_ antagonist) the response to ET-1.

Because the model parameters were optimized to fit both studies simultaneously, some aspects of the experimental data are fit less than perfectly. The optimization process makes tradeoffs between individual study and variable fits to find the set of parameters that best fits the data overall. For instance, the observed RBF response to ET-1 infusion in (Figure 4A) was stronger than the observed response to ET-1 infusion in (Figure 5 - yellow), even though the increase in plasma ET-1 was slightly higher in Bohm et al. Thus, the optimization produced a simulated change in RBF that was slightly weaker than observed in the first study and slightly stronger than observed in the second study. The mechanistic effects of ET-1 infusion, adjusted to reproduce the outcomes observed in Kaasjager et al. (1997) are depicted in Figure 4B.

**FIGURE 6:**
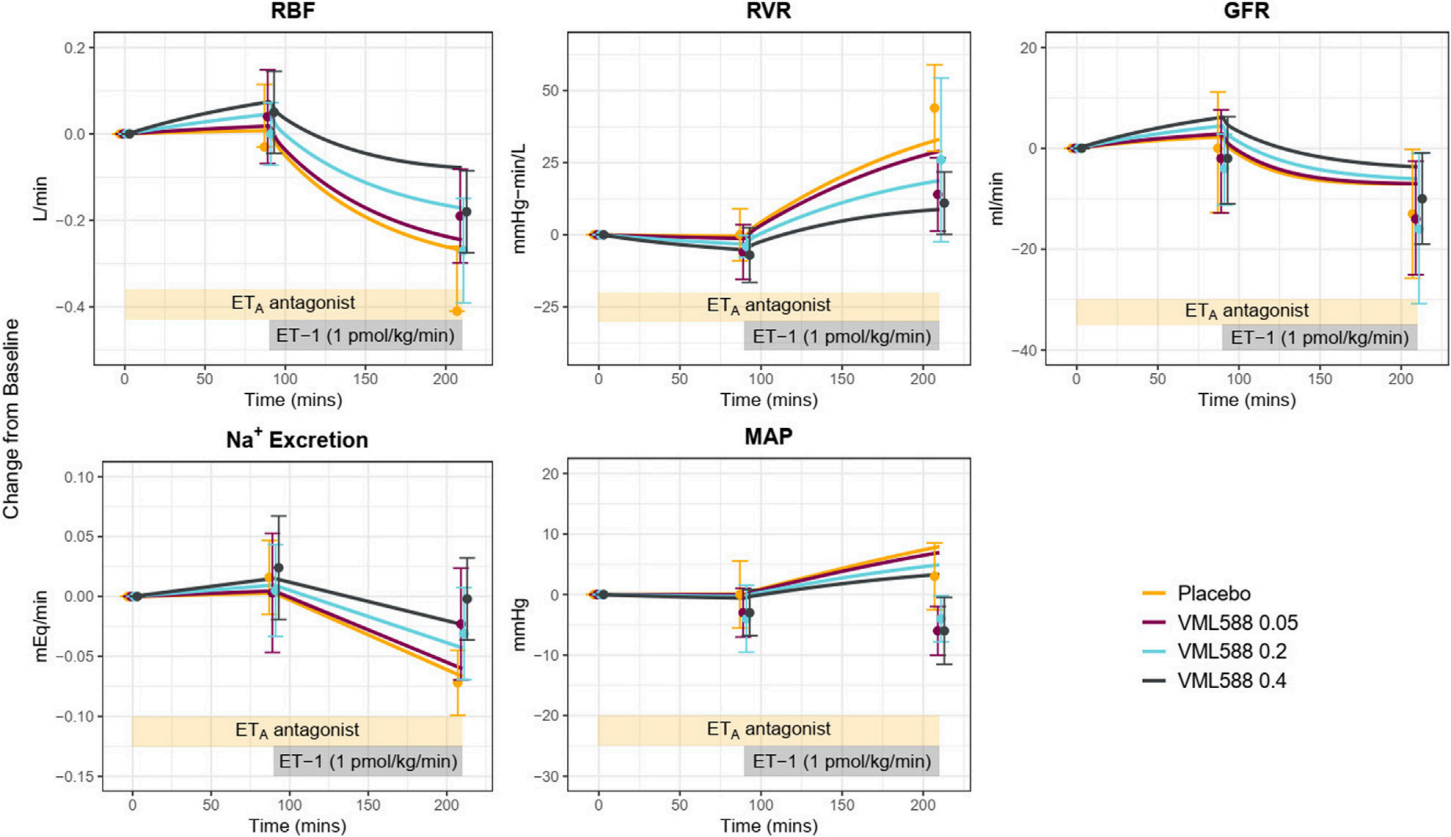
Model validation: Using identified mechanisms and calibrated parameters, the model reasonably reproduces the response to ET_A_ antagonism (VML588) and ET-1 infusion in a separate clinical study in healthy subjects (Vuurmans et al., 2004). RBF, renal blood flow; RVR, renal vascular resistance; GFR, glomerular filtration rate; MAP, mean arterial pressure.

Using the calibrated parameters, the model reasonably predicted the response to the ET_A_ antagonist VML588, as shown in Figure 6. To simulate this study, only the plasma concentrations of VMK 588 were adjusted–all other parameters were fixed to their estimated values in Table 1. The model reproduced observed changes in GFR and Na^+^ excretion in response to ET_A_ inhibition well, alone and with ET-1 infusion. It also reproduced the changes in RBF and RVR, although the predicted response was on the low end of the standard error of the measured value. For MAP, the model reproduced the lack of change with ET_A_ inhibition alone (at 90 min), and the simulated rise in MAP with ET-1 infusion at 210 min fell within the standard error of the measured value, although it was on the high end.

However, while it reproduced the trend of a reduction in MAP with ET_A_ antagonism relative to placebo during ET-1 infusion, the simulated absolute MAP at 210 min fell above the observed values in the ET_A_ antagonist arms. This is likely due to differences in the observed MAP response to ET_A_ antagonism between the calibration study (Bohm et al., 2003) and the experimental study used for validation. In MAP remained unchanged during ET-1 infusion following ET_A_ antagonism (Figures 5 blue), while in MAP fell below baseline during ET-1 infusion and ET_A_ antagonism. Increasing the simulated concentration of VML588 (and thus the degree of ET_A_ inhibition) could improve the MAP response but worsened the response of other variables (not shown).

This validation step demonstrated that the calibrated model and mechanisms identified can reasonably predict the key trends and behaviors in a new study. But this new study also provides further information for further constraining the model parameters. Therefore, the model parameters were estimated again, this time including the data from Vuurmans et al. (2004) in the objective function. The parameter estimates from the initial and refined calibration are given in Table 1. Parameter values shifted slightly from the initial calibration, but there were no major changes in values.

### 3.2 ET-1 mechanisms

#### 3.2.1 Renal vascular effects

The strongest and most important mechanism of ET-1 identified was a vasoconstrictive effect through ET_A_ on the renal preglomerular vasculature (preafferent and afferent arterioles). This effect was identified in the first round of optimization and greatly reduced the objective function relative to the NULL model, and to a vastly greater extent than other mechanisms tested. After including this mechanism, though, other mechanisms provided substantial further improvements in the model. On the efferent arteriole, a weak vasoconstrictive effect of ET-1 through ET_A_ and a vasodilatory effect through ET_B_ were found to be important, but these effects were much weaker than the preglomerular effect of ET_A_. No effect of ET_B_ on the afferent arteriole was necessary to explain the data.

These findings are generally consistent with the experimental literature. The renal vasoconstrictive effects of ET-1 are well-established (Kohan et al., 2011), and ET_A_ expression has been found in all parts of the renal vasculature (Davenport et al., 1994; Endlich et al., 1996; Wendel et al., 2006). However, it is expressed relatively higher in the preglomerular vasculature (Wendel et al., 2006; Kohan et al., 2011; Davenport et al., 2016). Studies have shown that ET_A_ antagonists reduce vasoconstriction of the preafferent and afferent arterioles with ET-1 infusion (Endlich et al., 1996; Inscho et al., 2005), and the maximum vasoconstrictive effect of ET-1 on the afferent is greater than on efferent (Edwards et al., 1990). Thus, the finding of a strong vasoconstrictive effect of ET_A_ on the afferent and weaker effect on the efferent is consistent with these studies.

Studies in the hydronephrotic rat kidney have reported that ET_A_ antagonists block preglomerular constriction with ET-1, but have little effect on efferent tone (Endlich et al., 1996). Experiments in blood-perfused juxtaglomerular nephron preparations found that ET_B_ constricts the afferent arteriole but dilates the efferent arteriole. In this study, the vasodilatory effect of ET_B_ on efferent resistance was detected, although it was the least necessary to explain the data. An effect of ET_B_ on afferent resistance was not detected. This does not necessarily conflict with the experiments by (Inscho et al., 2005)—but it suggests that the data used in building this model was not sufficient to detect this mechanism, and suggests that this effect is less important in determining the response to ET-1 infusion as ET_A_/ET_B_ agonists under the conditions in the calibration experiments.

#### 3.2.2 Systemic arterial vasoconstriction

**FIGURE 7:**
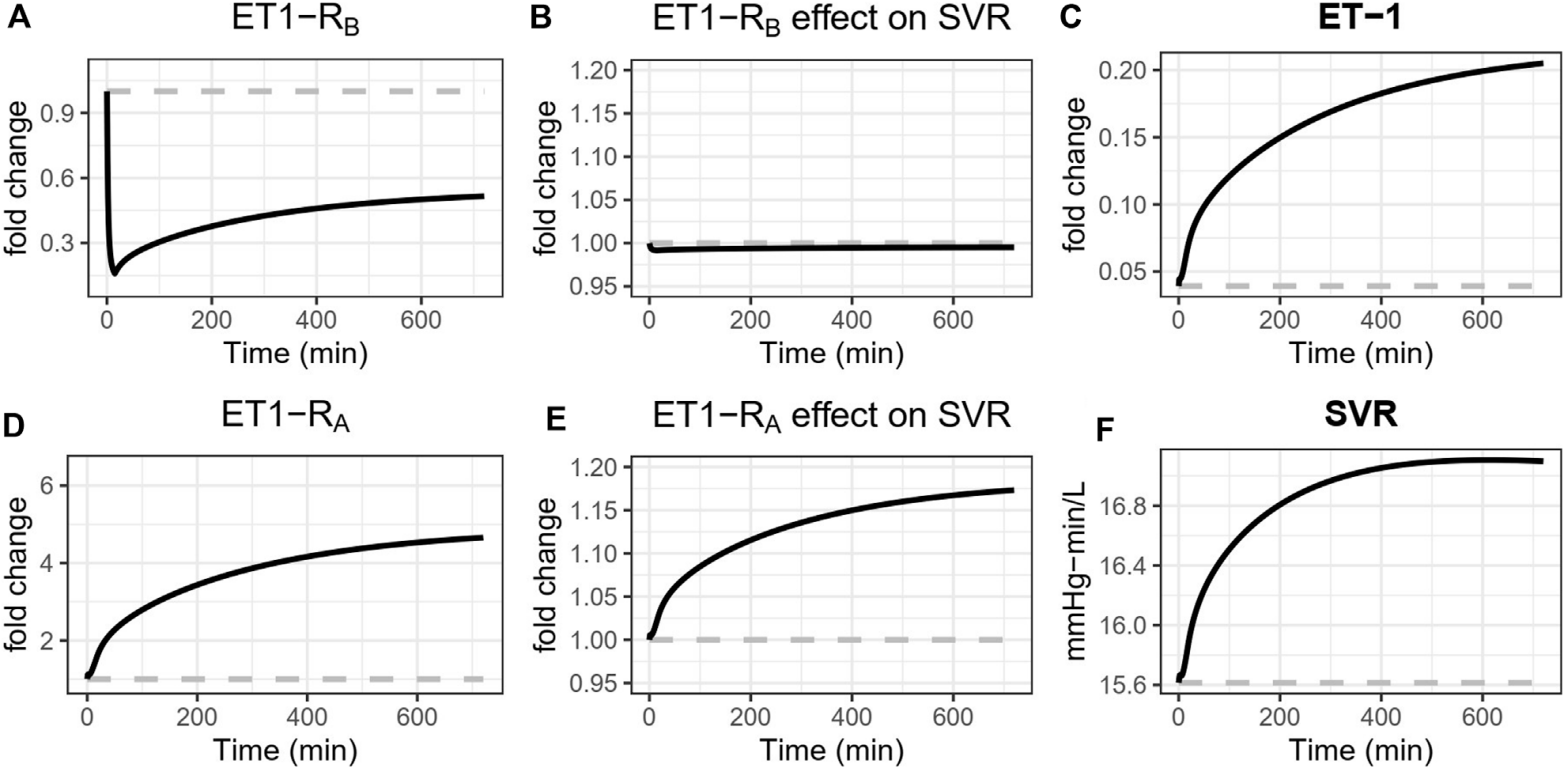
Simulated effects of ETB antagonism with BQ788 on systemic vascular resistance (SVR). ET_B_ antagonism reduces ET-1 binding to ET_B_ **(A)**, sending a weak vasodilatory signal to SVR **(B)**. But because ET_B_ is the main clearance receptor for ET-1, ET_B_ antagonism also causes ET-1 to rise **(C)**, thus increasing its binding to the ET_A_ receptor **(D)**. Because the vasoconstrictive effect of ET_A_ is much stronger than that of ET_B_, the vasoconstrictive effect through ET_A_ dominates **(E)**, causing SVR to rise **(F)**.

A vasoconstrictive effect of both ET_A_ and ET_B_ on the systemic vasculature was identified, and the effect through ET_A_ was about four times stronger than the effect through ET_B_. The vasoconstrictive effect of ET-1 through ET_A_ on a wide range of blood vessel types is well established (Davenport et al., 2016). However, the data regarding ET_B_ is conflicting. Of particular interest, while studies have found that ET_B_ antagonists induce constriction (Love et al., 2000), studies of the ET_B_ agonist sarafotoxin have found that it also induces constriction (Haynes et al., 1995). These results at first seem in conflict, but the model is actually consistent with both of these results and offers an explanation as well. This is illustrated in Figure 7, which shows the simulated changes in systemic vascular resistance (SVR), [ET-1], [ET1-RA], [ET1-RB], and their respective effects on vascular resistance during ET_B_ antagonism. Because ET_B_ stimulates vasoconstriction, ET_B_ antagonism reduces ET-1 binding to ET_B_, sending a weak vasodilatory signal to SVR. But because ET_B_ is the main clearance receptor for ET-1, ET_B_ antagonism also causes ET-1 to rise, thus increasing its binding to the ET_A_ receptor. Because the vasoconstrictive effect of ET_A_ is much stronger than that of ET_B_, the vasoconstrictive effect through ET_A_ dominates, causing SVR to rise. A similar effect occurs to renal vascular resistance.

#### 3.2.3 Sodium transport

The second most important effect in explaining the experimental data, after the ET_A_ vasoconstriction of the preglomerular vasculature, was an effect of ET-1 on sodium retention in the proximal tubule through ET_A_. ET_A_ is expressed in the proximal nephron, and studies that have measured lithium clearance (a measure of proximal sodium reabsorption) with ET-1 infusion have consistently found a decrease in lithium clearance or fractional excretion of lithium, indicating an increase in proximal Na + reabsorption (Rabelink et al., 1994; Sorensen et al., 1994; Kaasjager et al., 1997; Vuurmans et al., 2004). However, studies of ET-1 control of sodium excretion are complex and difficult to study at the organ level, and results across studies are conflicting (Kohan et al., 2011). Garcia and Garvin found increased PT fluid reabsorption at low ET-1 concentrations (0.1–1 p.m.) and decreased reabsorption at high concentrations (∼1,000 p.m.) (Garcia and Garvin, 1994). ET-1 concentrations in the experimental studies used in fitting the model ranges from 1 to 50 p.m., closer to the low-concentration range used by Garcia and Garvin, and thus consistent with sodium retention.

Effects of ET_B_ on sodium transport, in either the proximal tubule or the collecting duct, were found to be unnecessary to explain the experimental data. This does not mean that this effect does not exist–experimental studies have demonstrated a role of ET_B_ in collecting duct natriuresis (Kohan et al., 2011). However, it indicates that this effect cannot be detected in the data used for calibration, and that this mechanism is not necessary to explain the responses observed in the experimental studies considered. In (Kaasjager et al., 1997), the decrease in fractional excretion of lithium parallels the changes in Na + excretion, and the effects of ET-1 on proximal tubule reabsorption are sufficient to produce the observed Na + excretion rates in this study, as well as in the validation study by (Vuurmans et al., 2004).

#### 3.2.4 Venous constriction/reduced venous capacitance

The model was insensitive to effects of ET-1 on venous capacitance or venous compliance. Including this effect tended to shift other parameters, but did not improve or worsen the objective function. This indicates that the measured data does not hold sufficient information to identify and quantify venous effects. However, the effects of ET-1 on venous tone through ET-1 have been clearly demonstrated experimentally. ET-1 caused both venous and arterial contractions in both human and canine vessels, with significantly lower EC50 in veins compared to arteries (Cocks et al., 1989). Maximum contraction in veins was 100% that of max contraction with K+ depolarization, while in arteries it ranges from 25% to 80%. In small arteries and veins, ET_A_ antagonists blocked this effect, but ET_B_ antagonists and agonists had no effect, indicating that it is mediated by ET_A_ (Riezebos et al., 1994). Therefore, further investigating and additional data is needed to better inform this mechanism in the model going forward.

#### 3.2.5 The role of ET_B_

ET_B_ antagonism induces renal vasoconstriction and reduced renal blood flow (see Figure 5), but interestingly, the only identified direct effects of ET_B_ were weak systemic vasoconstriction and weak efferent vasodilation. The model suggests that the effects of ET_B_ antagonists are primarily the consequence of reduced clearance of ET-1 through ET_B_ when it is blocked, resulting in higher plasma and renal ET-1 and increased binding to the ET_A_ receptor (Figure 7). In the context of ET_A_ antagonist selectivity, this suggests that as selectivity decreases and the potential for ET_B_ binding increases, the primary consequence is likely to be reduced ET-1 clearance, increased ET-1 concentrations, more ET-1 available to bind to any open ET_A_ receptors, thus effectively reducing the degree of ET_A_ antagonism.

#### 3.2.6 Limitations

There are a number of limitations of this study. As noted, the ability to detect ET-1 mechanisms is limited by the data used to inform the model. Lack of identification of an effect does not mean an effect does not exist. It only means that the effect is not necessary to explain the observed data, and mechanisms not detected in this study may emerge as important if additional variables were measured. For example, effects on venous capacitance were not needed o explain the current data, but this could be because the data utilized included only measures that strongly reflect arterial function (e.g., cardiac output and blood pressure). Inclusion of additional variables such as venous pressure or cardiac filling pressure may be necessary to identify a venous effect, but these variables are unfortunately much more difficult to obtain clinically.

This model provides a starting point for continuous testing and integration of additional data sets going forward, which may allow detection and quantification of further mechanisms, especially in the collecting duct and venous circulation. Also, inclusion of additional data sets may allow identification of nonlinear effects, which could not be detected in this study.

All experimental studies used in this analysis were conducted in men. Therefore, this model represents the male response to ET-1. The response could look distinctly different in females, and studies conducted in females should be incorporated into the model in the future.

## 4 Conclusion

In this study, we updated our previously published cardiorenal model to account for the pathophysiological mechanism of ET1 and its complexes of ET1A and ET1B. The physiologic mechanisms of ET-1 through each of its receptors in the systemic and renal vasculature and renal tubules was rigorously evaluated and calibrated using clinical observations of acute vascular and renal response to ET-1 infusion and ET_A_/ET_B_ antagonists in healthy subjects. The model is capable of reproducing changes in blood pressure, renal blood flow, GFR, and sodium/water excretion with ET_A_ or ET_B_ antagonism. The mechanisms identified are consistent with the larger body of experimental studies on ET-1, and provide novel insights into the relative magnitude and importance of endothelin’s effects. The preglomerular vasoconstrictive effect of ET-1 through ET_A_ was found to be much stronger than either its efferent vasoconstrictive effect through ET_A_ or its efferent vasodilatory effect through ET_B_. This analysis suggests that the vasoconstrictive and fluid retention responses to ET_B_ antagonists are more likely explained by reduced ET-1 clearance by ET_B_, resulting in increased binding to ET_A_, rather than direct effects through ET_B_. However, finding that a mechanism was not necessary to explain the data in this analysis, which in included arterial and renal function measures, does not negate its existence. For instance, an effect on venous capacitance was not detected, but this could be due to lack of information on venous function in the variables measured. This model provides a tool for understanding and predicting clinical responses to therapeutics that targeting the endothelin system. For example, this model is currently being utilized to aid in the clinical development of the highly selective ET_A_ antagonist zibotentan by predicting the renal hemodynamics and fluid status alone and in combination with a sodium glucose cotransporter 2 (SGLT2 inhibitor).

## Data Availability

The original contributions presented in the study are included in the article/Supplementary Material, further inquiries can be directed to the corresponding author.
